# From trial to population: a study of a family-based community intervention for childhood overweight implemented at scale

**DOI:** 10.1038/ijo.2014.103

**Published:** 2014-07-29

**Authors:** J Fagg, P Chadwick, T J Cole, S Cummins, H Goldstein, H Lewis, S Morris, D Radley, P Sacher, C Law

**Affiliations:** 1 Centre for Paediatric Epidemiology and Biostatistics, Institute of Child Health, University College London, London, UK; 2 Department of Clinical, Educational and Health Psychology, University College London, London, UK; 3 Department of Social and Environmental Health Research, London School of Hygiene and Tropical Medicine, London, UK; 4 Centre for Multilevel Modelling, University of Bristol, Bristol, UK; 5 Psychosocial and Family Services, Great Ormond Street Hospital for Children NHS Foundation Trust, London, UK; 6 Department of Applied Health Research, University College London, London, UK; 7 Carnegie Faculty, Leeds Metropolitan University, Leeds, UK; 8 Childhood Nutrition Research Centre, Institute of Child Health, University College London, London, UK

## Abstract

**Objectives:**

To assess how outcomes associated with participation in a family-based weight management intervention (MEND 7–13, Mind, Exercise, Nutrition..Do it!) for childhood overweight or obesity implemented at scale in the community vary by child, family, neighbourhood and MEND programme characteristics.

**Methods/Subjects:**

Intervention evaluation using prospective service level data. Families (*N*=21 132) with overweight children are referred, or self-refer, to MEND. Families (participating child and one parent/carer) attend two sessions/week for 10 weeks (*N*=13 998; *N*=9563 with complete data from 1788 programmes across England). Sessions address diet and physical activity through education, skills training and motivational enhancement. MEND was shown to be effective in obese children in a randomised controlled trial (RCT). Outcomes were mean change in body mass index (BMI), age- and sex-standardised BMI (*z*BMI), self-esteem (Rosenberg scale) and psychological distress (Strengths and Difficulties Questionnaire) after the 10-week programme. Relationships between the outcome and covariates were tested in multilevel models adjusted for the outcome at baseline.

**Results:**

After adjustment for covariates, BMI reduced by mean 0.76 kg m^−2^ (s.e.=0.021, *P*<0.0001), *z*BMI reduced by mean 0.18 (s.e.=0.0038, *P*<0.0001), self-esteem score increased by 3.53 U  (s.e.=0.13, *P*<0.0001) and psychological distress score decreased by 2.65 U (s.e.=0.31, *P*<0.0001). Change in outcomes varied by participant, family, neighbourhood and programme factors. Generally, outcomes improved less among children from less advantaged backgrounds and in Asian compared with white children. BMI reduction under service conditions was slightly but not statistically significantly less than in the earlier RCT.

**Conclusions:**

The MEND intervention, when delivered at scale, is associated with improved BMI and psychosocial outcomes on average, but may work less well for some groups of children, and so has the potential to widen inequalities in these outcomes. Such public health interventions should be implemented to achieve sustained impact for all groups.

## Introduction

Childhood overweight (including obesity) is prevalent in many countries^1^ and associated with poorer physical and psychosocial health across the life course.^2,3^ Furthermore, overweight is not distributed evenly across the population and varies by ethnicity, socioeconomic circumstances, gender and age.^4,5^ The high prevalence and associated burden of childhood overweight necessitates treatment as well as prevention.

A recent Cochrane review and meta-analysis of family-based interventions targeting overweight or obese children concluded that such interventions may deliver ‘clinically relevant’ reductions in body mass index (BMI).^6^ However, most studies in this review were based on small, homogeneous samples and restricted research settings. This raises concerns about generalisability across all population groups (for example, low socioeconomic circumstances and minority ethnic groups) and implementation contexts, leaving the questions of ‘what works for whom and in what circumstances?’ largely unanswered.^6^ This is important because interventions have the potential to maintain, reduce or generate health inequalities.^7^

Weight management interventions for children have been implemented widely across England,^8^ but there is little information about their performance in service settings. Adoption and implementation of these interventions at scale might be associated with loss of effectiveness.^9^ In addition, obesogenic environments implicated in the aetiology and maintenance of overweight^10^ may moderate the effects of interventions,^11^ but this has been little studied. We address these gaps using observational data from the MEND (Mind, Exercise, Nutrition..Do it!) 7-13 programme, a family-based community intervention implemented at scale under service conditions. We assess whether the biological and psychosocial outcomes associated with participation in the intervention differ by participant, family, programme and neighbourhood characteristics. We also compare changes in BMI observed under service conditions with those observed under research conditions.

## Materials and methods

MEND 7–13 is a multi-component family-based community intervention that aims to support families of overweight or obese children to adopt and sustain healthier lifestyles. The intervention addresses diet and physical activity through education, skills training and motivational enhancement. Because of the importance of family involvement for behaviour change, the intervention requires a parent or carer to attend all 20 sessions (over 10 weeks). The MEND 7–13 intervention was developed to be delivered in community settings such as schools or leisure centres^12^ and delivered by a wide range of health, physical activity and social care professionals. Children are eligible if they are between 7 and 13 years old and overweight or obese (hereafter referred to as overweight, defined as exceeding the 91st centile of the UK 1990 BMI reference). MEND 7–13 was demonstrated in a randomised controlled trial (RCT) to be effective in reducing BMI of obese children at 6 months from baseline.^12^

Between 2007 and 2010, the MEND 7–13 intervention was implemented on a large scale, with MEND programmes (hereafter ‘programmes’) rolled out across all regions of England. The intervention was delivered by local community-based ‘delivery partner’ organisations. Intervention content and training were provided to delivery partners by MEND Central, a social enterprise.

Delivery partners recorded attendance of participants at each session, and measured height and weight to the nearest 0.1 cm and 0.1 kg using electronic scales following standardised procedures.

Self-esteem was reported by participants on a modified Rosenberg Self-Esteem scale.^13^ Designed for adolescents, the 10 scale items^13^ were modified to suit the younger age group (for example, wording such as ‘satisfied’ was clarified with ‘happy’ in brackets). Responses were on a four-point agree–disagree scale (coding in brackets); a lot like me (0), a bit like me (1), not like me (2) and not at all like me (3).

Participant psychological distress was reported by parents on the Strengths and Difficulties questionnaire (SDQ).^14^ The score comprises 25 items making up five subscales: peer problems, conduct, hyperactivity, anxiety and pro-social behaviour.

Parents also reported the participant’s ethnicity (white, Asian, black or other) and family socioeconomic circumstances including: family structure (lone parent/carer or couple parents/carers); housing tenure (owner occupied, social rented or private rented); and employment status of the ‘primary earner’ (employed or unemployed).

Delivery partners recorded data in an online database collated by MEND Central. For this study, a copy of the database for the period January 2007 to December 2010 was transferred to UCL Institute of Child Health (ICH) for analysis. The UCL Ethics Committee granted approval for the study in October 2010 (REF: 2677/002).

Height and weight data were cleaned to remove implausible values (those exceeding 7 s.d. from the mean and further outliers identified graphically). We calculated BMI (weight height^−2^) and its derived *z*-score (*z*BMI), standardised for age and sex using the UK 1990 BMI growth reference.^15,16^ Self-esteem items were coded and summed as recommended,^13^ a high value indicating high self-esteem (score range=0–30). Total psychological distress was calculated following authors’ guidelines by summing twenty items (the pro-social subscale is not included); a high value indicated high psychological distress (score range =0–40).^14^

For our analyses, outcomes were change in BMI, *z*BMI, self-esteem and SDQ, calculated as baseline (first session of the programme) subtracted from follow-up (penultimate (19th) session). Therefore, negative values for change indicated a fall in BMI and *z*BMI; a fall in self-esteem (Rosenberg self-esteem); and a fall in psychological distress (SDQ).

Participants’ residential postcodes were assigned Lower Super Output Area (LSOA) codes, representing small areas with a mean population of 1500 across England. LSOA codes were then used to attach a measure of neighbourhood deprivation (deciles of the Income Deprivation Affecting Children Index (IDACI) 2007),^17^ urban/rural status (urban, suburban or rural),^18^ the density of local fast food outlets per LSOA^19^ and the built environment (based on factor analysis of the percentage of the LSOA made up of roads and green space).^20^ We counted how many children attended each programme at baseline (hereafter referred to as ‘programme group size’) and the number of programmes that a local programme manager had managed as at the start of each programme. Approximately 80% of measured heights were rounded to whole or half centimetres. We derived a variable indicating if more than 20% of the height measures for a programme were rounded and included this in models to adjust for possible effects of data quality. We also derived a similar measure for weight rounding, where values were rounded to the nearest 0.5 kg. We categorised those attending fewer than 25% of sessions as non-completers, 25–75% as partial completers and more than 75% as completers.

Data were imputed for ethnicity (33% missing), family structure (36%), housing tenure (35%), employment status (63%) and percentage of sessions attended (42%). A multilevel (participants nested in programmes) multiple imputation model (*N*=13 998) was used to adjust for between-programme variation in missingness in MEND 7–13 programmes. The model assumed that data were missing at random—that missingness on variables was associated with other variables included in the multiple imputation model. Ten imputed data sets were produced and analysis results were combined using Rubin’s rules.^21^ We followed the guidelines of Sterne *et al.*^22^ for the analysis and reporting of missing data and multiple imputation (available on request). To test whether our findings were influenced by using imputed data, we also conducted sensitivity analyses, including analysis using complete case data with and without the variable describing parental employment status, as missingness was relatively high for this variable (data provided in Supplementary Information).

We also used unpublished data from participants in the intervention arm (*N*=47) of the RCT of MEND 7–13^12^ to compare change in BMI under trial and service conditions. Height and weight were measured in the first and penultimate sessions of the trial as in the service data. Age, sex, baseline BMI, ethnicity and housing tenure were also measured.

**Figure 1:**
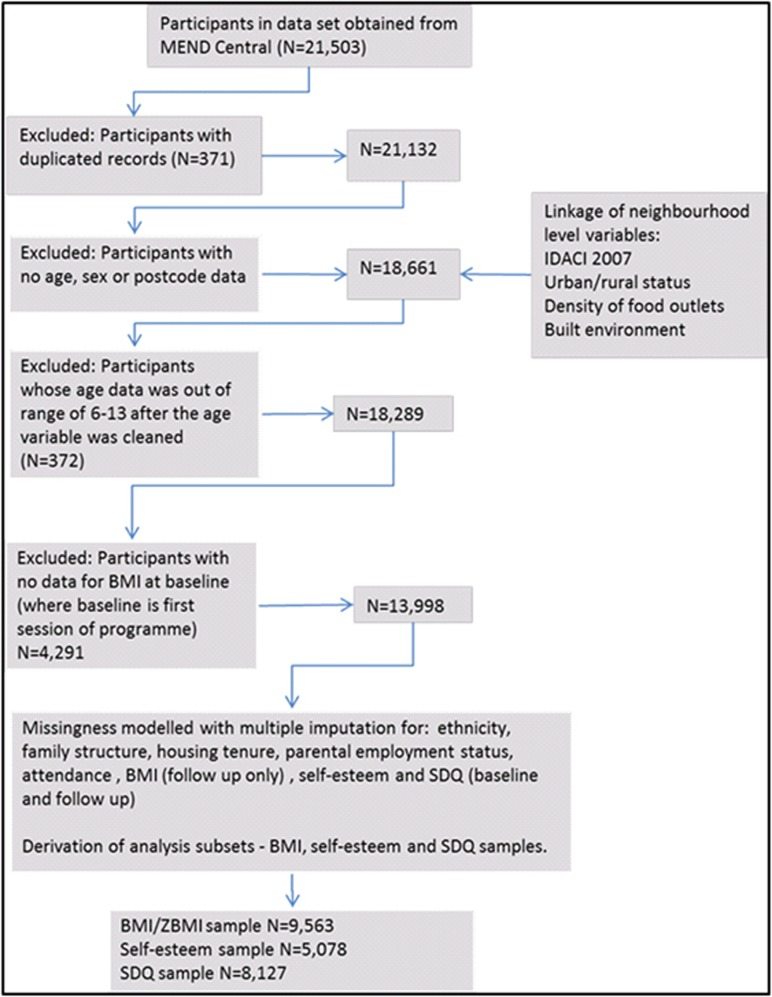
Flow chart of referral to MEND 7–13 and data management.

Following the Sterne guidelines,^22^ analysis outcomes were included in the multiple imputation model where they were missing to ensure that covariates were imputed correctly. However, analysis data sets excluded cases where outcomes were not completely observed at both baseline and follow-up. Sample sizes of the four data sets for analysis of change in BMI, *z*BMI, self-esteem and SDQ, respectively, are given in Figure 1.

Four sets of two-stage analyses were conducted, one for each outcome. In the first stage, relationships between the outcome and each covariate were tested in multilevel models adjusted for the outcome measured at baseline (‘baseline-adjusted’ models). If the relationship between the covariate and the outcome was statistically significant, the covariate was carried forward to a multilevel multivariable model. The intercept of the multivariable model describes the mean change in the outcome for a given ‘reference group’, which for categorical variables was the largest group, whereas for continuous variables were grand mean centred (allowing the intercept of the model to be interpreted as the mean change). Coefficients in the model describe the amount and direction of change per unit change in the covariates, relative to the reference group.

The random intercept terms estimate variations in outcomes between participants and between programmes. Random slopes were also assessed for age, sex and ethnicity to examine whether the random intercept varied by those factors. *A priori*-specified interaction terms were also tested for: each outcome at baseline and age, sex and ethnicity, for lone parent family status and the built environment, and for age and sex. Models with random slopes or interaction terms were judged an improvement on models with no additional terms if the Bayesian Information Criterion was more than four points smaller.^23^

Change in BMI in the service data was also compared with the RCT data, with the service data for this analysis being restricted to obese children to match the RCT. This multilevel model was based on complete case data, adjusted for covariates measured in both data sets (age, sex, ethnicity and housing tenure), to account for potential differences in sample composition.

The multilevel multiple imputation model was estimated using REALCOM-IMPUTE.^24^ All analyses were conducted using Stata version 12.1 software,^25^ and multilevel models were fitted in MLwiN ^26^ using the Stata programme runmlwin.^27^ Statistical significance was set at the 5% level.

## Results

Families (21 132) were referred to the intervention, of which 18 289 had complete data for age, sex and residential postcode (Figure 1). Of these, 13 998 attended a MEND 7–13 programme, 9563 had complete data for change in BMI and *z*BMI (‘BMI sample’), 5078 had complete data for change in self-esteem (‘self-esteem sample’) and 8127 had complete data for change in SDQ (‘SDQ sample’).

Descriptive statistics (Table 1) were estimated using the BMI sample. As statistics were similar for the self-esteem and SDQ samples, they are not reported here (available on request). Most participants were obese rather than overweight, exceeding the 98th UK 1990 centile. The average age of participants was 10, there were more girls than boys and most children were white. Two-thirds of parents were couples, over half were owner occupiers and three-quarters of households had an employed primary earner. Compared with all LSOAs in England, families lived in LSOAs that were: more income deprived (England mean IDACI 2007=0.21); more likely to be urban (England urban LSOAs=80.6%); and more built up (England built environment score mean=0); but similar in terms of fast food outlet density (England 5+ outlets per LSOA=8%). The mean number of programmes managed previously by programme managers was six programmes, while mean programme group size was nine participants. Three-quarters completed >75% of sessions. Most programmes rounded 20% or more of height measures, whereas just over half rounded 20% or more of weight.

The density of local fast food outlets per LSOA and height/weight rounding variables were not associated with change in BMI and *z*BMI after adjustment for baseline values (data not shown). In addition, sex and the number of programmes per programme manager were not associated with change in *z*BMI. Other covariates were statistically significant and retained for multivariable models. *A priori*-specified interaction terms and random slopes did not improve fit and so these parameters were not retained in the BMI and *z*BMI models.

Change in self-esteem and SDQ were not associated with age, family structure, housing tenure, urban/rural status, programme group size or weight rounding after adjustment for baseline values. Change in self-esteem was also not associated with the built environment, sex or height rounding, whereas change in SDQ was also not associated with employment status (data not shown). Other covariates were statistically significant and retained for multivariable models as was a random slope for parental employment status in the SDQ model.

In the multivariable model, BMI in the reference group fell on average by 0.76 kg m^−2^ (Table 2, model 1). In absolute terms, mean BMI fell in all subgroups. Relative to the reference group, BMI fell more for children who were male and with higher baseline BMI; and less for those who were older, from Asian or Black ethnic groups (compared with white groups), living with unemployed (rather than employed) primary earners, living in more deprived neighbourhoods, participating in larger programme groups and partial- and non-completers rather than completers. In the multivariable model, *z*BMI fell by 0.18 U  (Table 2, model 2). Results were similar to those for change in BMI, except that *z*BMI fell less for children with a higher baseline *z*BMI.

Self-esteem rose on average by 3.53, approximately half a s.d. of baseline self-esteem (Table 3, model 1), and increased across all subgroups. In relative terms, self-esteem increased less for children with higher baseline self-esteem, for children from Asian ethnic groups versus white children and for partial completers versus completers. SDQ fell on average by 2.65, a third of a s.d. of baseline SDQ (Table 3, model 2), and fell across all subgroups. In relative terms, SDQ reduced more for children with higher baseline SDQ at baseline, for Black compared with white children and those attending programmes with rounded height data; and less for boys, children living in more income-deprived neighbourhoods, where the programme manager had delivered more programmes, non-completers and partial completers rather than completers.

Sensitivity analyses showed that, in general, the direction and order of magnitude of coefficients that were significant in the models estimated using imputed data were similar in those estimated using complete case data (see Supplementary Information for data). However, the material loss of power led to some coefficients being estimated as non-significant in the complete case analyses.

The reduction in BMI for 8–12 year old obese children was 0.79 kg m^−2^ (95% CI (confidence interval)=0.74, 0.84) in the service data compared with 1.04 kg m^−2^ (95% CI=0.79, 1.29) in the RCT (adjusted for baseline BMI, age, sex, ethnicity and housing tenure). This difference was not statistically significant.

## Discussion

We found that a family-based community intervention for childhood overweight or obesity, when implemented at scale and under service conditions, was associated with improvements in BMI and in psychosocial outcomes. The reduction in BMI under service conditions was slightly but not statistically significantly less than that observed in the RCT of the same intervention. Although previous research has shown that family-based interventions for child overweight are associated with changes in adiposity^6^ and psychosocial^28^ outcomes when tested under trial conditions, to our knowledge this is the first study to show that such interventions implemented at scale and under service conditions might also be associated with changes in these outcomes.

Our results also showed that all population subgroups improved on average for all outcomes, but that improvements varied by participant, family, programme and neighbourhood factors. For example, BMI fell more in children with higher baseline BMI, or who were younger, male, white, from families with an employed primary earner or who lived in less deprived areas. BMI also fell more if the child attended more programme sessions and if the programme group was relatively small, suggesting a ‘dose’ effect. Increase in self-esteem was less for children with high baseline self-esteem, Asian children and partial completers. SDQ fell more in children with high baseline SDQ, Black compared with white children and for participants attending programmes where height data were rounded. SDQ reductions were smaller for boys, children living in more deprived neighbourhoods, children participating in programmes where the programme manager had delivered more programmes, non-completers and partial completers.

Our findings therefore show that the intervention, although benefiting all groups to some extent, may also have the potential to widen existing ethnic^4^ and socioeconomic^5^ inequalities in childhood overweight and psychosocial outcomes. Such findings may provide the potential for developing programmes such as MEND (for example, by modifying content, training and implementation) to make them more successful for groups who currently respond less well to the intervention.

Although an obesogenic environment is thought to promote the development and maintenance of childhood overweight,^29^ little work has assessed whether weight management interventions are moderated by features of the wider environment. We found that the outcomes associated with the MEND intervention did not vary with urban/rural characteristics or indicators of the food and built environment. However, measures of area deprivation did appear to moderate changes in BMI and SDQ associated with the intervention, independent of individual socioeconomic circumstances, and this may be capturing unmeasured environmental characteristics that impair successful weight management.

This analysis was based on a large individual-level data set collected under service conditions across all regions of England. It allowed us to estimate with adequate statistical power how outcomes of the MEND intervention varied by participant, family, neighbourhood and programme characteristics, estimates that would be underpowered in most research studies. However, in the absence of controls, such associations cannot be equated with effectiveness. We were able to compare the size of change in BMI in the service data with that observed in the RCT, and they were similar, but we had limited power to assess differences between them (there were only 47 children in the intervention arm of the RCT^12^). The data we analysed were collected for service provision and not for research. We used a range of techniques for improving data quality, including algorithms for data cleaning, and multiple imputation for missing data. These techniques were aimed at maximising the value of the observed data and minimising bias. We fitted models using imputation and complete case approaches and found that, other than the differences in statistical significance that are to be expected given the greater power of imputation models, findings were similar. Nevertheless, some bias may still be present.

There is little research on what happens once interventions found to be effective in a research setting are implemented in practice at scale.^30^ We demonstrate here that the MEND intervention when delivered at scale is associated with improved BMI and psychosocial outcomes on average, while at the same time having the potential to widen inequalities in these outcomes. We do not know to what extent our findings can be generalised to other weight management programmes or to other community-based interventions. However, our findings suggest that implementation of such interventions should be accompanied by evaluation not only of sustained impact but also of equality of impact at both the individual and population level.

There is little consensus about what constitutes a clinically significant reduction in BMI^31^ or how much average BMI would need to be reduced in the population of overweight children to reduce the population-level burden of childhood overweight. Further research should clarify these questions. In addition, data from longer-term follow-up were not available following the service intervention (follow-up in the RCT was to 1 year) and so the estimates derived here cannot be used to comment on whether improvements in BMI and other outcomes were sustained beyond the end of the programme when delivered in service settings.

## Figures and Tables

**Table 1:** Descriptive statistics of MEND 7–13 participants

*Variables*	*BMI sample (*N*=9563)*	
	*%/mean*	*s.d. for means*
*Outcomes at baseline*		
% Obese (BMI exceeds 98th centile)	84.3	
BMI baseline in kg m^−2^ (mean, s.d.)	26.8	4.65
* z*BMI baseline (mean, s.d.)	2.7	0.72
Self-esteem baseline (mean, s.d.)^a^	16.8	6.85
SDQ baseline (mean, s.d.)^b^	13.1	6.87

*Covariates*		
Age in years (mean, s.d.)	10.4	1.75
Sex (girls:boys, %)	55.3:44.7	
Ethnicity (%)		
White	77.3	
Asian	12.5	
Black	6.2	
Other	3.9	
Family structure (couple:lone, %)	67.2:32.8	
* *Housing tenure (%)		
Owner occupied	56.4	
Social rented	29.4	
Private rented	14.2	
Employment status (employed:unemployed, %)	76.7:23.3	
Neighbourhood deprivation, IDACI 2007 (mean, s.d.)	0.26	0.19
Urban/rural (%)		
Urban	88.5	
Towns	6.9	
Villages	4.6	
Built environment (mean, s.d.)	0.18	0.93
Density of local fast food outlets per LSOA		
None	46.3	
1–2 Unhealthy outlets	33.2	
3–4 Unhealthy outlets	12.4	
5+ Unhealthy outlets	8.1	
Number of programmes per PM^c^ (mean, s.d.)	5.8	5.72
Programme group size (mean, s.d.)	8.6	3.09
Sessions attended (%)		
Non-completers	2.1	
Partial completers	25.8	
Completers	72.1	
Height rounding (not rounded:rounded, %)	3.7:96.3	
Weight rounding (not rounded:rounded, %)	41.3:58.7	

Abbreviations: BMI, body mass index; IDACI, Income Deprivation Affecting Children Index; LSOA, Lower Super Output Area; ref., reference category; SDQ, Strengths and Difficulties questionnaire; *z*BMI, BMI-derived *z*-score.

a Calculated from imputed self-esteem data set (*N*=5078).

b Calculated from imputed SDQ data set (*N*=8127).

c PM, programme manager.

**Table 2:** Regression coefficients (s.e.) for change in BMI and *z*BMI, at the participant, family, programme and neighbourhood level from multivariable models

*Parameters*	*Change in BMI (*N*=9563)*			*Change in* z*BMI (*N*=9563)*		
	*B*	*s.e.*	P*-value*	*B*	*s.e.*	P*-value*
*Fixed part*						
Intercept	−0.76	0.021	<0.0001	−0.18	0.0038	<0.0001
BMI baseline	−0.022	0.0020	<0.0001	0.029	0.0024	<0.0001
Age	0.018	0.0054	0.00089	0.015	0.00099	<0.0001
Sex (ref. girls)						
Boys	−0.085	0.017	<0.0001	—	—	—
Ethnicity (ref. white)						
Asian	0.15	0.037	<0.0001	0.029	0.0074	0.0001
Black	0.15	0.040	0.00023	0.022	0.0079	0.0056
Other	0.088	0.046	0.055	0.017	0.0090	0.056
Family structure (ref. couple)						
Lone parent	0.011	0.020	0.58	0.0033	0.0040	0.41
Housing tenure (ref. owner occupied)						
Social rented	0.020	0.024	0.40	0.0081	0.0047	0.086
Private rented	0.0085	0.033	0.80	0.0031	0.0067	0.65
Parental employment (ref. employed)						
Unemployed	0.06	0.026	0.021	0.0099	0.0050	0.048
IDACI 2007	0.15	0.061	0.012	0.034	0.012	0.0044
Built environment	0.012	0.013	0.37	0.0017	0.0026	0.51
Urban/rural status (ref. urban)						
Towns	−0.033	0.040	0.40	−0.0082	0.0078	0.29
Villages	−0.0071	0.047	0.89	−0.00010	0.0092	0·99
Number of programmes per PM^a^	0.0033	0.0020	0.10	—	—	—
Programme group size	0.0087	0.0039	0.025	0.0018	0.00077	0.019
Attendance (ref. completer)						
Non-completer	0.21	0.074	0.0040	0.034	0.015	0.020
Partial completer	0.13	0.027	<0.0001	0.023	0.0051	<0.0001
						
*Random part*						
Between programmes	0.11	0.008	<0.0001	0.0045	0.00032	<0.0001
Between participants	0.63	0.010	<0.0001	0.024	0.00039	<0.0001

Abbreviations: BMI, body mass index; IDACI, Income Deprivation Affecting Children Index; ref., reference category; *z*BMI, BMI-derived *z*-score.

a PM, programme manager.

**Table 3:** Regression coefficients (s.e.) for change in self-esteem and SDQ, at the participant, family, programme and neighbourhood level from multivariable models

*Parameters*	*Change in self-esteem (*N*=5078)*			*Change in SDQ (*N*=8127)*		
	B	*s.e.*	P*-value*	B	*s.e.*	P*-value*
*Fixed part*						
Intercept	3.53	0.13	<0.0001	−2.65	0.31	<0.0001
Self-esteem/SDQ baseline	−0.41	0.012	<0.0001	−0.34	0.0073	<0.0001
Sex (ref. girls)						
Boys	—	—	—	0.54	0.099	<0.0001
Ethnicity (ref. white)						
Asian	−0.72	0.28	0.0094	0.053	0.20	0.80
Black	0.16	0.34	0.64	−0.50	0.22	0.024
Other	−0.42	0.39	0.29	−0.26	0.26	0.31
Parental employment (ref. employed)						
Unemployed	−0.30	0.26	0.25	0.091	0.16	0.56
IDACI 2007	−0.85	0.49	0.085	1.04	0.31	0.00086
Built environment	—	—	—	0.041	0.062	0.50
Number of programmes per PM^a^	0.0074	0.029	0.80	0.022	0.0094	0.019
Height rounding						
Rounded	—	—	—	−0.84	0.30	0.0045
Attendance (ref. completer)						
Non-completer	−0.47	0.72	0.51	1.05	0.49	0.033
Partial completer	−0.53	0.23	0.024	0.43	0.14	0.0021
						
*Random part*						
Between programme	1.79	0.37	<0.0001	0.87	0.21	<0.0001
Between programme (covariance)	—	—	—	−0.14	0.38	0.70
Between programme (unemployed)	—	—	—	2.60	0.93	0.0054
Between participants	30.8	0.68	<0.0001	18.00	0.34	<0.0001

Abbreviations: IDACI, Income Deprivation Affecting Children Index; ref., reference category; SDQ, Strengths and Difficulties questionnaire.

a PM, programme manager.
