# Supplementary material for: From trial to population: a study of a family-based community intervention for childhood overweight implemented at scale
Source: Int J Obes (Lond). 2014 Jul 29;38(10):1343–9. doi: 10.1038/ijo.2014.103 (PMC4175967; doi:10.1038/ijo.2014.103)
Supplement: Supplementary Information [file ijo2014103x1.doc]

**Supplement to Fagg et al. submission to the International Journal of Obesity 2014: sensitivity analyses**

Introduction

This supplement refers to an analysis of how the outcomes of a childhood weight management intervention delivered under service conditions vary by participant, family, neighbourhood, and programme factors as described in a paper submitted to the International Journal of Obesity (*“*From trial to population: a study of a family-based community intervention for childhood overweight implemented at scale*”*). This supplement assumes that the reader has read the article to which this document refers and therefore does not define terminology or explain analyses which are described in that article.

Data analysed in the paper were collected by the staff delivering the intervention. In line with other service level and routine data sources, the data were subject to relatively extensive missingness. To minimise bias, adjust for multilevel missingness, and maximise power, we imputed data using a multilevel multiple imputation model. To test whether our findings were influenced by using imputed data, we present sensitivity analyses for all four outcomes referred to in the paper (change in BMI, zBMI, self-esteem and SDQ) using analysis based on complete case data, including and excluding employment status.

**Table 1: Regression coefficients (standard error) for change in BMI, at the participant, family, programme and neighbourhood level from multivariable models using imputed (N=9 563), complete case (N=2 150) and complete case data excluding employment status (N= 3** 986)

|  | **Imputed** | | |  | **Complete case** | | |  | **Complete case (excl. employment)** | | |
| --- | --- | --- | --- | --- | --- | --- | --- | --- | --- | --- | --- |
| **Parameters** | **B** | **(se)** | **P** |  | **B** | **(se)** | **p** |  | **B** | **(se)** | **p** |
| **Fixed part** |  |  |  |  |  |  |  |  |  |  |  |
| Intercept | -0.76 | (0.021) | <0.0001 |  | -0.80 | (0.036) | <0.0001 |  | -0.77 | (0.027) | <0.0001 |
| BMI baseline | -0.022 | (0.0020) | <0.0001 |  | -0.03 | (0.0042) | <0.0001 |  | -0.027 | (0.0031) | <0.0001 |
| Age | 0.018 | (0.0054) | 0.00089 |  | 0.021 | (0.011) | 0.052 |  | 0.021 | (0.0082) | 0.012 |
| Sex (ref. Girls) |  |  |  |  |  |  |  |  |  |  |  |
| Boys | -0.085 | (0.017) | <0.0001 |  | -0.06 | (0.035) | 0.085 |  | -0.07 | (0.026) | 0.0082 |
| Ethnicity (ref. white) |  |  |  |  |  |  |  |  |  |  |  |
| Asian | 0.15 | (0.037) | <0.0001 |  | 0.20 | (0.065) | 0.0021 |  | 0.22 | (0.05) | <0.0001 |
| Black | 0.15 | (0.040) | 0.00023 |  | 0.10 | (0.072) | 0.16 |  | 0.092 | (0.053) | 0.084 |
| Other | 0.088 | (0.046) | 0.055 |  | 0.11 | (0.083) | 0.20 |  | 0.064 | (0.061) | 0.30 |
| Family structure (ref. Couple) |  |  |  |  |  |  |  |  |  |  |  |
| Lone parent | 0.011 | (0.020) | 0.58 |  | -0.0084 | (0.041) | 0.84 |  | 0.0017 | (0.03) | 0.95 |
| Housing tenure (ref. Owner Occupied) |  |  |  |  |  |  |  |  |  |  |  |
| Social rented | 0.020 | (0.024) | 0.40 |  | 0.004 | (0.049) | 0.94 |  | 0.062 | (0.035) | 0.078 |
| Private rented | 0.0085 | (0.033) | 0.80 |  | -0.063 | (0.057) | 0.27 |  | -0.0096 | (0.042) | 0.82 |
| Parental employment (ref. Employed) |  |  |  |  |  |  |  |  |  |  |  |
| Unemployed | 0.06 | (0.026) | 0.021 |  | 0.16 | (0.049) | 0.0011 |  | Excluded | | |
| IDACI 2007 | 0.15 | (0.061) | 0.012 |  | 0.055 | (0.12) | 0.66 |  | 0.14 | (0.09) | 0.13 |
| Built environment | 0.012 | (0.013) | 0.37 |  | 0.022 | (0.026) | 0.40 |  | 0.0036 | (0.02) | 0.86 |
| Urban/rural status (ref. Urban) |  |  |  |  |  |  |  |  |  |  |  |
| Towns | -0.033 | (0.040) | 0.40 |  | -0.14 | (0.082) | 0.094 |  | -0.09 | (0.062) | 0.15 |
| Villages | -0.0071 | (0.047) | 0.89 |  | -0.0015 | (0.089) | 0.99 |  | 0.024 | (0.068) | 0.73 |
| Number of programmes per PM~ | 0.0033 | (0.0020) | 0.10 |  | 0.0067 | (0.003) | 0.027 |  | 0.004 | (0.0026) | 0.13 |
| Programme group size | 0.0087 | (0.0039) | 0.025 |  | 0.013 | (0.0072) | 0.074 |  | 0.0081 | (0.0055) | 0.14 |
| Attendance (ref. Completer) |  |  |  |  |  |  |  |  |  |  |  |
| Non-completer | 0.21 | (0.074) | 0.0040 |  | -0.022 | (0.24) | 0.93 |  | 0.46 | (0.15) | 0.0017 |
| Partial completer | 0.13 | (0.027) | <0.0001 |  | 0.23 | (0.049) | <0.0001 |  | 0.29 | (0.038) | <0.0001 |
| **Random part** |  |  |  |  |  |  |  |  |  |  |  |
| Between programmes | 0.11 | (0.008) | <0.0001 |  | 0.096 | (0.015) | <0.0001 |  | 0.084 | (0.011) | <0.0001 |
| Between participants | 0.63 | (0.010) | <0.0001 |  | 0.58 | (0.02) | <0.0001 |  | 0.62 | (0.016) | <0.0001 |

~ PM – Programme manager

**Table 2: Regression coefficients (standard error) for change in zBMI, at the participant, family, programme and neighbourhood level from multivariable models using imputed (N=9 563), complete case (N=2 150) and complete case data excluding employment status (N= 3** 986)

|  | **Imputed** | | |  | **Complete case** | | |  | **Complete case (excl. employment)** | | |
| --- | --- | --- | --- | --- | --- | --- | --- | --- | --- | --- | --- |
| **Parameters** | **B** | **(se)** | **P** |  | **B** | **(se)** | **p** |  | **B** | **(se)** | **p** |
| **Fixed part** |  |  |  |  |  |  |  |  |  |  |  |
| Intercept | -0.18 | (0.0038) | <0.0001 |  | -0.18 | (0.0069) | <0.0001 |  | -0.18 | (0.0049) | <0.0001 |
| zBMI baseline | 0.029 | (0.0024) | <0.0001 |  | 0.021 | (0.0049) | <0.0001 |  | 0.022 | (0.0036) | <0.0001 |
| Age | 0.015 | (0.00099) | <0.0001 |  | 0.012 | (0.0020) | <0.0001 |  | 0.013 | (0.0015) | <0.0001 |
| Ethnicity (ref. white) |  |  |  |  |  |  |  |  |  |  |  |
| Asian | 0.029 | (0.0074) | <0.0001 |  | 0.043 | (0.014) | 0.0017 |  | 0.044 | (0.0099) | <0.0001 |
| Black | 0.022 | (0.0079) | 0.0056 |  | 0.014 | (0.015) | 0.35 |  | 0.015 | (0.011) | 0.16 |
| Other | 0.017 | (0.0090) | 0.056 |  | 0.019 | (0.017) | 0.29 |  | 0.014 | (0.012) | 0.24 |
| Family structure (ref. Couple) |  |  |  |  |  |  |  |  |  |  |  |
| Lone parent | 0.0033 | (0.0040) | 0.41 |  | -0.00054 | (0.0086) | 0.95 |  | 0.0033 | (0.0059) | 0.58 |
| Housing tenure (ref. Owner Occupied) |  |  |  |  |  |  |  |  |  |  |  |
| Social rented | 0.0081 | (0.0047) | 0.086 |  | 0.0060 | (0.010) | 0.56 |  | 0.013 | (0.0069) | 0.065 |
| Private rented | 0.0031 | (0.0067) | 0.65 |  | -0.010 | (0.012) | 0.38 |  | -0.00058 | (0.0082) | 0.94 |
| Parental employment (ref. Employed) |  |  |  |  |  |  |  |  |  |  |  |
| Unemployed | 0.0099 | (0.0050) | 0.048 |  | 0.024 | (0.010) | 0.018 |  | Excluded | | |
| IDACI 2007 | 0.034 | (0.012) | 0.0044 |  | 0.013 | (0.026) | 0.62 |  | 0.032 | (0.018) | 0.068 |
| Built environment | 0.0017 | (0.0026) | 0.51 |  | 0.0032 | (0.0055) | 0.57 |  | -0.00035 | (0.0039) | 0.93 |
| Urban/rural status (ref. Urban) |  |  |  |  |  |  |  |  |  |  |  |
| Towns | -0.0082 | (0.0078) | 0.29 |  | -0.015 | (0.017) | 0.40 |  | -0.0092 | (0.012) | 0.46 |
| Villages | -0.00010 | (0.0092) | 0.99 |  | -0.00044 | (0.019) | 0.98 |  | 0.0059 | (0.013) | 0.66 |
| Programme group size | 0.0018 | (0.00077) | 0.019 |  | 0.0029 | (0.0015) | 0.053 |  | 0.0019 | (0.0011) | 0.085 |
| Attendance (ref. Completer) |  |  |  |  |  |  |  |  |  |  |  |
| Non-completer | 0.034 | (0.015) | 0.020 |  | -0.013 | (0.049) | 0.80 |  | 0.064 | (0.029) | 0.026 |
| Partial completer | 0.023 | (0.0051) | <0.0001 |  | 0.042 | (0.010) | <0.0001 |  | 0.048 | (0.0075) | <0.0001 |
| **Random part** |  |  |  |  |  |  |  |  |  |  |  |
| Between programmes | 0.0045 | (0.00032) | <0.0001 |  | 0.0043 | (0.00069) | <0.0001 |  | 0.0037 | (0.00045) | <0.0001 |
| Between participants | 0.024 | (0.00039) | <0.0001 |  | 0.025 | (0.00087) | <0.0001 |  | 0.024 | (0.00061) | <0.0001 |

~ PM – Programme manager

**Table 3: Regression coefficients (standard error) for change in self-esteem, at the participant, family, programme and neighbourhood level from multivariable models using imputed (N=5 078), complete case (N=1 852) and complete case data excluding employment status (N= 2** 393)

|  | **Imputed** | | |  | **Complete case** | | |  | **Complete case (excl. employment)** | | |
| --- | --- | --- | --- | --- | --- | --- | --- | --- | --- | --- | --- |
| **Parameters** | **B** | **(se)** | **p** |  | **B** | **(se)** | **p** |  | **B** | **(se)** | **p** |
| **Fixed part** |  |  |  |  |  |  |  |  |  |  |  |
| Intercept | 3.53 | (0.13) | <0.0001 |  | 3.20 | (0.19) | <0.0001 |  | 3.31 | (0.16) | <0.0001 |
| Self-esteem baseline | -0.41 | (0.012) | <0.0001 |  | -0.42 | (0.018) | <0.0001 |  | -0.41 | (0.017) | <0.0001 |
| Ethnicity (ref. white) |  |  |  |  |  |  |  |  |  |  |  |
| Asian | -0.72 | (0.28) | 0.0094 |  | -0.56 | (0.45) | 0.21 |  | -0.59 | (0.42) | 0.16 |
| Black | 0.16 | (0.34) | 0.64 |  | 0.017 | (0.5) | 0.97 |  | -0.18 | (0.47) | 0.70 |
| Other | -0.42 | (0.39) | 0.29 |  | 0.28 | (0.6) | 0.65 |  | 0.07 | (0.54) | 0.90 |
| Parental employment (ref. Employed) |  |  |  |  |  |  |  |  |  |  |  |
| Unemployed | -0.30 | (0.26) | 0.25 |  | -0.078 | (0.31) | 0.80 |  | Excluded | | |
| IDACI 2007 | -0.85 | (0.49) | 0.085 |  | -1.33 | (0.79) | 0.092 |  | -0.99 | (0.7) | 0.16 |
| Programme group size | 0.0074 | (0.029) | 0.80 |  | 0.0031 | (0.046) | 0.95 |  | 0.0069 | (0.043) | 0.87 |
| Attendance (ref. Completer) |  |  |  |  |  |  |  |  |  |  |  |
| Non-completer | -0.47 | (0.72) | 0.51 |  | -1.86 | (2.06) | 0.37 |  | -0.3 | (1.5) | 0.84 |
| Partial completer | -0.53 | (0.23) | 0.024 |  | -0.015 | (0.36) | 0.97 |  | -0.42 | (0.34) | 0.22 |
| **Random part** |  |  |  |  |  |  |  |  |  |  |  |
| Between-programme | 1.79 | (0.37) | <0.0001 |  | 1.85 | (0.61) | 0.0025 |  | 2.1 | (0.58) | 0.0003 |
| Between-participants | 30.8 | (0.68) | <0.0001 |  | 27.8 | (1.04) | <0.0001 |  | 30.9 | (1) | <0.0001 |

**Table 4: Regression coefficients (standard error) for change in SDQ, at the participant, family, programme and neighbourhood level from multivariable models using imputed (N=8 127), complete case (N=1 918) and complete case data excluding employment status (N= 3 576)**

|  | **Imputed** | | | |  | **Complete case** | | |  | **Complete case (excl. employment)** | | |
| --- | --- | --- | --- | --- | --- | --- | --- | --- | --- | --- | --- | --- |
| **Parameters** | **B** | **(se)** | | **p** |  | **B** | **(se)** | **p** |  | **B** | **(se)** | **p** |
| **Fixed part** |  |  | |  |  |  |  |  |  |  |  |  |
| Intercept | -2.65 | (0.31) | | <0.0001 |  | -2.49 | (0.40) | <0.0001 |  | -2.61 | (0.38) | <0.0001 |
| SDQ baseline | -0.34 | (0.0073) | | <0.0001 |  | -0.34 | (0.014) | <0.0001 |  | -0.33 | (0.011) | <0.0001 |
| Sex (ref. girls) |  |  | |  |  |  |  |  |  |  |  |  |
| Boys | 0.54 | (0.099) | | <0.0001 |  | 0.16 | (0.20) | 0.43 |  | 0.28 | (0.15) | 0.063 |
| Ethnicity (ref. white) |  |  | |  |  |  |  |  |  |  |  |  |
| Asian | 0.053 | (0.20) | | 0.80 |  | 0.26 | (0.36) | 0.47 |  | 0.019 | (0.27) | 0.94 |
| Black | -0.50 | (0.22) | | 0.024 |  | -0.28 | (0.40) | 0.48 |  | -0.57 | (0.29) | 0.054 |
| Other | -0.26 | (0.26) | | 0.31 |  | -0.31 | (0.48) | 0.53 |  | -0.43 | (0.34) | 0.21 |
| Parental employment (ref. Employed) |  |  |  | |  |  |  |  |  |  |  |  |
| Unemployed | 0.091 | (0.16) | | 0.56 |  | -0.15 | (0.30) | 0.61 |  | Excluded | | |
| IDACI 2007 | 1.04 | (0.31) | | 0.00086 |  | 1.40 | (0.64) | 0.028 |  | 0.99 | (0.45) | 0.028 |
| Built env. | 0.041 | (0.062) | | 0.50 |  | 0.048 | (0.13) | 0.70 |  | -0.044 | (0.093) | 0.64 |
| Programmes per PM | 0.022 | (0.0094) | | 0.019 |  | 0.024 | (0.015) | 0.10 |  | 0.021 | (0.013) | 0.12 |
| Height rounding |  |  | |  |  |  |  |  |  |  |  |  |
| Rounded | -0.84 | (0.30) | | 0.0045 |  | -0.73 | (0.37) | 0.047 |  | -0.67 | (0.36) | 0.062 |
| Attendance |  |  | |  |  |  |  |  |  |  |  |  |
| Non-completer | 1.05 | (0.49) | | 0.033 |  | 1.99 | (1.53) | 0.19 |  | 1.16 | (0.87) | 0.18 |
| Partial completer | 0.43 | (0.14) | | 0.0021 |  | 0.091 | (0.28) | 0.75 |  | 0.28 | (0.21) | 0.19 |
| **Random part** |  |  | |  |  |  |  |  |  |  |  |  |
| Between-programme | 0.87 | (-0.21) | | <0.0001 |  | 1.11 | (0.43) | 0.010 |  | 1.37 | (0.28) | <0.0001 |
| Between-programme (covariance) | -0.14 | (-0.38) | | 0.70 |  | -0.79 | (0.82) | 0.33 |  | Excluded | | |
| Between-programme (unemployed) | 2.60 | (-0.93) | | 0.0054 |  | 7.47 | (2.15) | 0.0005 |  | Excluded | | |
| Between-participants | 18.00 | (-0.34) | | <0.0001 |  | 16.7 | (0.66) | <0.0001 |  | 18.1 | (0.48) | <0.0001 |

~ PM – Programme manager
